# Factors Affecting Mortality in Patients Admitted to the Hospital by Emergency Physicians despite Disagreement with Other Specialties

**DOI:** 10.1155/2020/2173691

**Published:** 2020-03-13

**Authors:** Engin Ozakin, Arif Alper Cevik, Filiz Baloglu Kaya, Nurdan Acar, Fikri M. Abu-Zidan

**Affiliations:** ^1^Department of Emergency Medicine, Eskisehir Osmangazi University, College of Medicine and Health Sciences, Eskişehir, Turkey; ^2^Departments of Internal Medicine, Emergency Medicine Section, College of Medicine and Health Sciences, United Arab Emirates University, Al Ain, UAE; ^3^Department of Surgery, College of Medicine and Health Sciences, United Arab Emirates University, Al Ain, UAE

## Abstract

**Background:**

Emergency physicians (EPs) face critical admission decisions, and their judgments are questioned in some developing systems. This study aims to define the factors affecting mortality in patients admitted to the hospital by EPs against in-service departments' decision and evaluate EPs' admission diagnosis with final discharge diagnosis.

**Methods:**

This is a retrospective analysis of prospectively collected data of ten consecutive years (2008–2017) of an emergency department of a university medical center. Adult patients (≥18 years-old) who were admitted to the hospital by EPs against in-service departments' decision were enrolled in the study. Significant factors affecting mortality were defined by the backward logistic regression model.

**Results:**

369 consecutive patients were studied, and 195 (52.8%) were males. The mean (SD) age was 65.5 (17.3) years. The logistic regression model showed that significant factors affecting mortality were intubation (*p* < 0.0001), low systolic blood pressure (*p* = 0.006), increased age (*p* = 0.013), and having a comorbidity (*p* = 0.024). There was no significant difference between EPs' primary admission diagnosis and patient's final primary diagnosis at the time of disposition from the admitted departments (*McNemar–Bowker test*, *p* = 0.45). 96% of the primary admission diagnoses of EPs were correct.

**Conclusions:**

Intubation, low systolic blood pressure on presentation, increased age, and having a comorbidity increased the mortality. EPs admission diagnoses were highly correlated with the final diagnosis. EPs make difficult admission decisions with high accuracy, if needed.

## 1. Introduction

Turkey is one of the leading countries in its region in implementing modern emergency care [1, 2]. It has the highest ED patient volume among countries in its region [3]. As a result, increased demand with inadequate resources caused admission difficulties. Although valuable improvements were achieved in all levels of healthcare, public demand on use of emergency departments (ED) is growing.

Furthermore, this is complicated by other factors including multiple patients' comorbidities, the idea of nonbenefit from admitting certain patients to the in-service units, resistance to take responsibility for complicated cases, noncompliance with universal admission criteria, and defensive medicine approaches [4–7]. The refusal to admit patients who need specialized care by in-service departments increases mortality [8–10]. Emergency physicians (EPs) have been given the legal responsibility by law to admit these patients when needed in Turkey [11]. This authorization is frequently questioned by other departments with a claim that EPs make unnecessary and wrong admissions.

We aimed to study the accuracy of EPs' diagnoses and to define the factors affecting mortality of patients admitted to the hospital by EPs against in-service departments' decision.

## 2. Materials and Methods

### 2.1. Ethical Approval

This study was reviewed and approved by the Research Ethics Committee of the College of Medicine of Eskisehir Osmangazi University (Approval Reference No. 2014-80558721/64).

### 2.2. Study Design and Setting

This is a retrospective analysis of prospectively collected data over ten consecutive years (2008–2017) from the Department of Emergency Medicine (EM) of Eskisehir Osmangazi University Medical Center. The ED treats around 115.000 emergency patients every year. The hospital is a tertiary care center having residency training programs and provides 24 hours emergency service for all specialties. It has 1200 beds including 120 intensive care unit beds.

### 2.3. Study Population

The studied patients were adults having an age of 18 years and more who were admitted to the hospital through the ED as decided by the emergency physicians (EPs) against the in-service departments' decision after being consulted. According to the National Emergency Department Management Legislation of Turkey, EPs have the responsibility to decide admissions in ambiguous cases who were not admitted by other departments [11]. This application has also been approved and applied by medical college/hospital councils. The patients who were transferred to other facilities or who left against medical advice after admission were excluded.

### 2.4. Decision-Making Process

**Figure 1:**
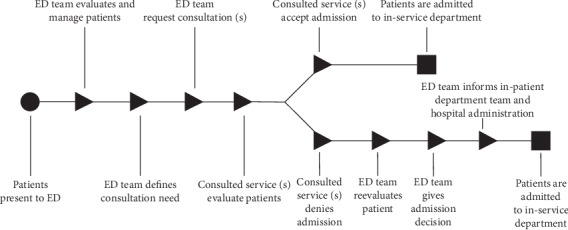
The decision-making process of patients' admission in the emergency department.

The decision-making process from presentation to admission is given in Figure 1. The patients who presented to the ED were initially evaluated and managed by the EM residents and consultants. EM senior residents who evaluated those cases were third or fourth-year residents. Consultant EPs were faculty members who have at least five years of clinical experience in tertiary care centers taking care of critically ill patients. Consultations for patients who needed hospital admission as judged by senior EM residents or consultants were requested. The senior residents (third or fourth year) or faculty members of the consulted specialty who have at least five years of clinical experience evaluated the patients. However, the consulted specialty admission or nonadmission decisions were always given by the faculty members of that specialty. If in-service departments denied patient admission, the patients were reevaluated by consultant EPs for a final decision. The consultant or senior resident of the in-patient service department was informed about ED admission decision directly by senior EM residents or consultant EPs. The hospital administrative officials were informed to facilitate the admission process. According to our national law, the care of these patients at this stage is the responsibility of the in-service department where the patients were admitted. The hospital administration was responsible for facilitating this process [11].

### 2.5. Data Collection

Data were extracted from the hospital information system and patients' files which were saved in ED archives. Patients' demography, presentation date and time, comorbidities, GCS, vital signs, presentation chief complaint, ED intubation, number of consultations, consulted department, ED length of stay, primary admission diagnosis, admitting location, hospital length of stay, transfer to other departments during admission, surgical operation, final disposition diagnosis, and 60 days in-hospital mortality were collected. The hospital uses the International Statistical Classification of Diseases and Related Health Problems 10th Revision (ICD-10) for coding the diagnoses. We accepted the final discharge diagnoses as the gold standard for the patients' diseases. The data accuracy was controlled by an emergency department faculty member for each case.

### 2.6. Statistical Analysis

The Mann–Whitney *U* test was used to compare continuous or ordinal data of two independent groups. Fisher's exact test was used to compare the categorical data of two independent groups. Spearman's correlation test was used to study the correlation between continuous data. The McNemar–Bowker test was used to compare dependent categorical data of two groups. Significant factors affecting mortality were defined by univariate analysis. The backward logistic regression model was used to define factors significantly affecting mortality. Factors that had a loose *p* value of 0.1 were entered into the model. The best cutoff point for predicting mortality was defined by a receiving operating characteristic (ROC) curve. PASW Statistics 21, SPSS Inc., USA, was used to analyze the data. A *p* value of less than 0.05 was accepted to be significant.

## 3. Results

**Figure 2:**
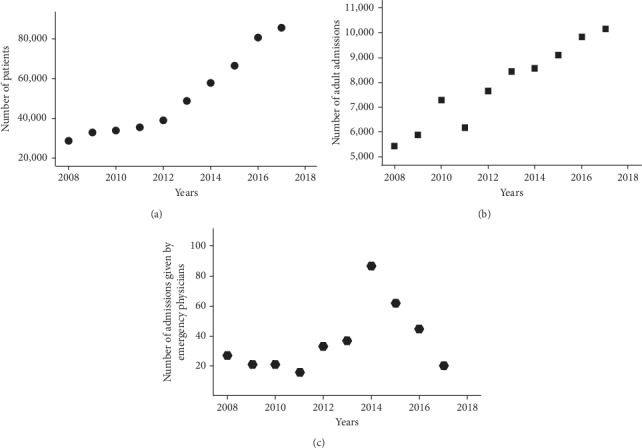
Annual total number of patients treated in the emergency department (a), admitted patients through the emergency department (b), and those admitted by emergency physicians against in-service departments' opinion (c).

A total of 509,408 adult patients were treated in the ED during the study period. 78,629 (15.4%) of them were admitted to the hospital through the ED during the same period. Three hundred sixty-nine patients (0.47%) were admitted to the hospital depending exclusively on EP decision, and 195 patients (52.8%) were males. The mean (SD) age of the patients was 65.5 (17.3) years. 134 (36.4%) patients died. There was an increase in the number of patients over time (Figures 2(a)–2(c)). There was a significant correlation between the number of treated and admitted patients (*Spearman's Rank Correlation*, *p* < 0.0001). The number of studied patients steadily increased till 2014 and then gradually decreased. The correlation between the number of studied patients and treated patients was not significant (*Spearman's Rank Correlation*, *p*=0.3). The correlation between studied and those admitted was also nonsignificant (*Spearman's Correlation*, *p*=0.21).

There was no difference between EPs' primary admission diagnoses and final diagnosis at discharge (*McNemar–Bowker test*, *p*=0.45). 95.9% of the primary admission diagnoses of EPs were the same as the final diagnoses. The admission location was changed by the in-service departments only in 25 patients (6.8%).

Significant factors affecting mortality in the univariate analysis (Tables 1 and 2) were age (*p*=0.001), GCS (*p* < 0.0001), systolic blood pressure (*p* < 0.0001), SpO_2_ (*p* < 0.0001), number of comorbidities (*p*=0.001), number of consultation (*p*=0.017), medical consultation (*p*=0.004), hospital stay (*p* < 0.0001), having a comorbidity (*p* < 0.0001), ED intubation (*p* < 0.0001), having a medico-legal case (*p*=0.001), admission location (*p*=0.005), and need for surgery (*p*=0.009). Year, month, weekdays, and presenting chief complaint were all nonsignificant and were not included in the tables (*p*=0.44, *p*=0.26, *p*=0.62, and *p*=0.17, respectively).

**Figure 3:**
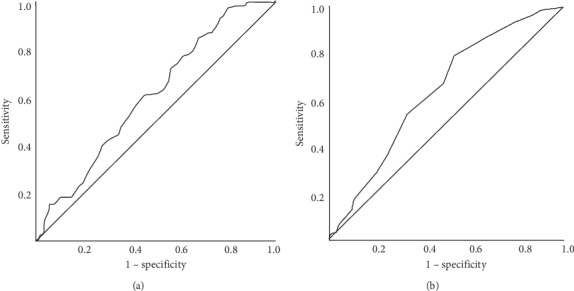
Receiver operating characteristic (ROC) curve defining the best cutoff point of age (a) and systolic blood pressure (b) that predicts death (a) and survival (b).

The backward logistic regression model defining factors affecting mortality was significant (Nagelkerke *R* Squared: 0.281, *p* < 0.0001) (Table 3). Significant factors in the model were ED intubation (*p* < 0.0001), systolic blood pressure (*p*=0.006), age (*p*=0.013), and having a comorbidity (*p*=0.024). One mmHg decrease in the systolic blood pressure increased the odds of death by 1%, and one-year increase in age increased the odds of death by 2% (Table 3). The areas under the curve for age and systolic blood pressure for predicting mortality were 60.3% and 64.1%, respectively (Figures 3(a) and 3(b)). An age of 65 had a sensitivity of 71.5% and a specificity of 43.8% for predicting mortality. A systolic blood pressure of 92 mmHg had a sensitivity of 79.1% and a specificity of 47% for predicting mortality.

## 4. Discussion

Our results showed that the EP primary diagnoses were highly accurate. Furthermore, the most significant factors affecting the 60-day in-hospital mortality in patients admitted by EPs despite disagreement with other specialties were ED intubation, low systolic blood pressure on presentation, increased age, and having a comorbidity.

EPs' diagnostic accuracy varied between 70 and 78% for medical cases and 80 and 96% for surgical cases [12–14]. Diagnostic accuracy is more difficult in the elderly (>65 years old) and nontrauma patients [12]. Our patients were mainly elderly having comorbidities. Chiu et al. found that diagnostic accuracy will improve with active observation and reassessment [12]. We think that the length of stay in our ED gave the opportunity for EPs to refine their final diagnosis.

Increased age, severity of the disease, comorbidities, bed shortage, high risk of mortality, and being too sick or well to benefit from admission are the factors causing admission reluctancy [10, 15, 16]. Patients who were refused to be admitted had double mortality compared with admitted patients [17]. Although there are universal admission guidelines to facilitate the admission decision for health professionals, the application of these guidelines vary between physicians [18, 19]. Depending on these guidelines, patients who were categorized as “too well to benefit” from admission had 17.6% in hospital mortality and 47% mortality at one-year follow-up [20].

Increased age affected the decision for hospital admission [9, 10, 21]. The proportion of elderly patients in the ED is higher than in adult and pediatric patients [22, 23]. Although the majority of these patients can be discharged, 20% of them could be admitted to the hospital because of critical problems in the following month [24]. Similar to our study, others showed that increased age increases mortality of patients who were refused admission [10, 25].

Increased comorbidity is associated with an increased risk of mortality [26]. There is a tendency by doctors to deny admission for patients having comorbidity because of increased risk of mortality [15]. This reflects the idea of “too sick to be benefited.” Furthermore, this attitude causes unnecessary delay and increased waiting times in the EDs. The median ED length of stay for those who died in our study was 380 minutes (>6 hours). Chalfin et al. reported that patients who were admitted from ED showed higher mortality if they stay for more than 6 hours in the ED [27]. Our study did not show a significant relation between length of stay and mortality, similar to others [28]. Nevertheless, others have shown the opposite [27, 29].

Low systolic blood pressure at admission increases mortality [21, 30, 31]. A single episode and prolonged hypotension (<100 mmHg) of nontraumatic ED patients is a significant contributing factor for unexpected in-hospital death [32]. There is a recent definition of hypotension in the elderly (less than 110 mmHg and even higher) [33, 34]. The cutoff levels of blood pressure affecting mortality was found to be 96 and 117 mmHg for the age groups of 36–64 and 65 and older, respectively [35]. Our study showed that the highest sensitivity and specificity for predicting mortality was a systolic blood pressure of 92 mmHg.

EPs perform multiple essential investigations and procedures during a shift. More critical procedures are needed for critical patients. Having a critical procedure in the ED is a major factor affecting patients' in-hospital outcome [27]. The environment of the ED is not strictly controlled as in the operating theatres or intensive care units. Accordingly, higher morbidity and mortality is expected in the ED in unstable patients [36]. Our study has shown that patients who were intubated in the ED had 67.4% in-hospital mortality.

### 4.1. Limitations

There are several limitations of our study. First, this study was performed in a single tertiary care center. Its results might not be generalizable to other settings. Second, the EPs who make admission decisions were academicians/core-faculty members of the EM residency program. Accordingly, the results of our study may not be generalized for less experienced EPs. Third, our medical care system relies on residents' involvement with every step of patient management, regardless of their training year. Depending on their experience, their communication with EPs consultants might affect admission decisions. Fourth, this study did not document the first working diagnosis of EPs before consultations. Accordingly, the primary admission diagnoses of EPs might have been affected by other specialties and discussions during the patients' ED stay. Fifth, because of the retrospective nature of the study, there might be other confounding factors affecting mortality which were not collected or found in the records. For example, the study possible failed to capture patients who may not have been refused but were strongly resisted by the in-patient service. This is shown by the low *R* square of the model (0.28). This indicates that the model can only explain 28% of the variance of the data. Sixth, the study group is a challenging, ambiguous, high-risk group of patients needing a higher level of clinical decision-making process and administrative applications which cannot be compared with simple direct pattern diagnosis and admissions. Finally, EPs and in-patient MDs may have been influenced by local training, incentives, or accountability, which are difficult to address in our current study without a well-defined accepted standard by the institution.

### 4.2. Future Directions

Answering the accuracy of EPs on admission decisions, defining and understanding the need for admission of patients according to universal guidelines or criteria, following the physician decision-making processes and their rationale behind admission or nonadmission decisions, and care or outcome differences between patients who were admitted without resistance and admitted depending on EPs' decision require a prospective, blind study. We hope researchers in the EM field will investigate this important practical issue that has not been sufficiently addressed before.

## 5. Conclusions

Our study has shown that being endotracheal intubation in the ED, having low systolic blood pressure, increased age, and comorbidities are the factors increasing in-hospital mortality, which is supported by the current literature. Furthermore, we found that EPs have high accuracy in their final diagnosis compared to other specialties and the in-service department where the patient should be admitted to. The decision to admit ambiguous, critically-ill patients to in-service departments can be challenging. However, EPs' critical admission decision-making for these high-risk patients is promising and should be supported by hospital administrations, especially in the institutions facing similar challenges.

## Figures and Tables

**Table 1:** Univariate analysis comparing continuous variables in patients who died and those who survived in the study population.

Variable	Survived (*n* = 235)	Died (*n* = 134)	*p* value
Age (years)	69 (17–99)	72 (18–76)	0.001
GCS	15 (3–15)	14 (3–15)	<0.0001
SBP (mmHg)	120 (0–260)	100 (0–200)	<0.0001
HR	105 (0–176)	105 (0–166)	0.35
RR	24 (0–60)	24 (0–52)	0.27
TEMP (°C)	36.4 (36–40.5)	36.4 (35–41.2)	0.38
SPO_2_ (%)	95 (40–100)	90 (0–99)	<0.0001
Number of comorbidities	1 (0–6)	2 (0–6)	0.001
Number of consultation	2 (1–7)	2 (1–7)	0.017
Medical consultation	2 (0–7)	2 (0–6)	0.004
Surgical consultation	0 (0–7)	1 (0–7)	0.8
Time in the ED (min)	440 (55–2230)	380 (120–1740)	0.1
Hospital stay (day)	10 (1–42)	4 (1–57)	<0.0001

Data are presented as the median (range) or number (percent) as appropriate. GCS : Glasgow coma scale; SBP : systolic blood pressure; HR : heart rate; RR: respiratory rate; TEMP : temperature; SPO_2_ : oxygen saturation; ED : emergency department.

**Table 2:** Univariate analysis comparing categorical variables in patients who died and those who survived in the study population.

Variable	Survived (*n* = 235)	Died (*n* = 134)	*p* value
Gender			0.52
Male	121 (51.5%)	74 (55.2%)	
Female	114 (48.5%)	60 (44.8%)	

Presentation time			0.14
08 : 00–16 : 00	107 (45.5%)	47 (35.1%)	
16 : 01–23 : 59	91 (38.7%)	63 (47.0%)	
00 : 00–07 : 59	37 (15.7%)	24 (17.9%)	

Comorbidity			<0.0001
Yes	172 (73.2%)	120 (89.6%)	
No	63 (26.8%)	14 (10.4%)	

Psychiatric illness			0.22
Yes	10 (4.3%)	2 (1.5%)	
No	225 (95.7%)	132 (98.5%)	

Consultation			<0.0001
Medical only	122 (51.9%)	66 (49.3%)	
Surgical only	45 (19.1%)	9 (6.7%)	
Both	68 (28.9%)	59 (44.0%)	

ED intubation			<0.0001
Yes	29 (12.3%)	60 (44.8%)	
No	206 (87.7%)	74 (55.2%)	

Medico-legal case			0.001
Yes	42 (17.9%)	8 (6.0%)	
No	193 (82.1%)	126 (94.0%)	

Admission location			0.005
ICU	183 (77.9%)	120 (89.6%)	
Ward	52 (22.1%)	14 (10.4)	

Surgical operation			0.009
Yes	19 (8.1%)	2 (1.5%)	
No	216 (91.9%)	132 (98.5%)	

Data are presented as the median (range) or number (percent) as appropriate.

**Table 3:** Backward logistic regression model defining significant factors affecting mortality (*n* = 369).

Variable	Estimate	SE	Wald test	*p* value	OR	95% CI
Intubation	1.67	0.29	32.74	<0.0001	5.33	3.01–9.45
SBP	−0.009	0.003	7.61	0.006	0.99	0.98–0.99
Age	0.022	0.009	6.17	0.013	1.02	1.01–1.04
Comorbidity	0.87	0.38	5.12	0.024	2.39	1.12–5.07
Constant	−2.08	0.73	8.08	0.004		

SE : standard error, OR : odds ratio, CI : confidence interval, SBP : systolic blood pressure.

## Data Availability

The data are available from the corresponding author upon appropriate request.

## Abbreviations

ED: Emergency department
EM: Emergency medicine
EPs: Emergency physicians
GCS: Glasgow coma scale
SBP: Systolic blood pressure.
